# Supplementary material for: Immune Cell Infiltration in the Microenvironment of Liver Oligometastasis from Colorectal Cancer: Intratumoural CD8/CD3 Ratio Is a Valuable Prognostic Index for Patients Undergoing Liver Metastasectomy
Source: Cancers (Basel). 2019 Dec 2;11(12):1922. doi: 10.3390/cancers11121922 (PMC6966431; doi:10.3390/cancers11121922)
Supplement: Supplementary file 1 [file cancers-11-01922-s001.pdf]

cancers

Supplementary materials

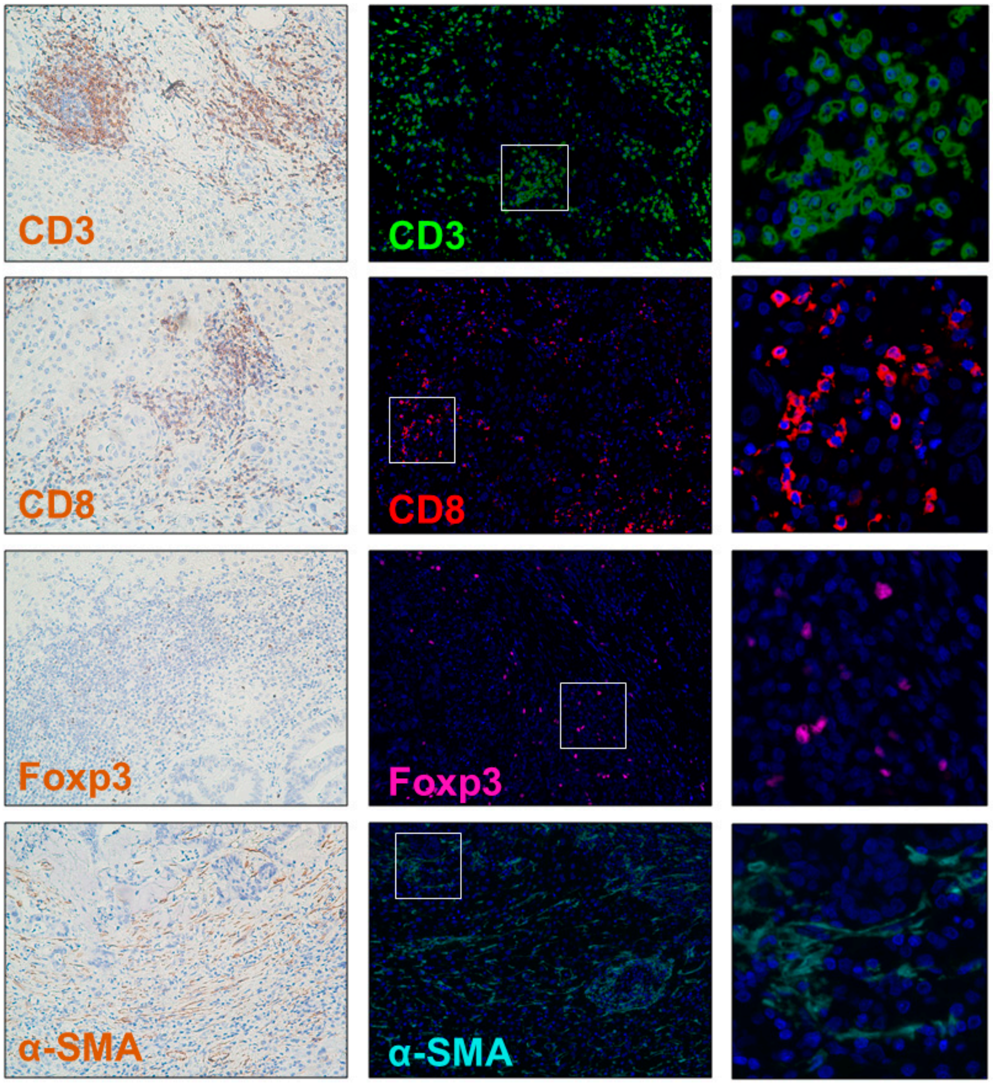

Figure S1. Microphotographs of representative examples of validation from IHC (left panels), uniplex IF immune cell markers or αSMA (middle panels), and details of uniplex IF in liver oligometastasis tissue (right panels).

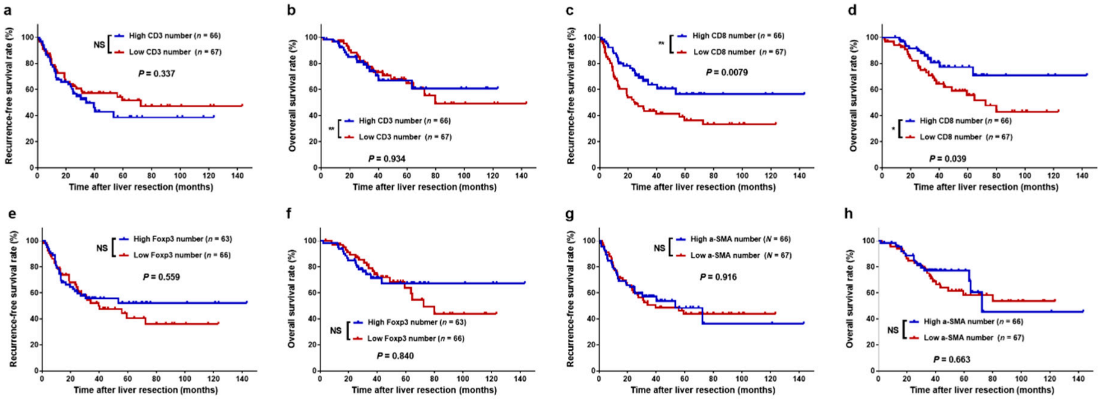

Figure S2. Kaplan–Meier survival curves of CLO patients after liver metastasectomy stratified by the different number of tumour-infiltrating lymphocyte and α-SMA positive cells in intratumoural regions of liver metastases. (a) Recurrence-free survival (RFS) for CD3 positive cells number; (b) overall survival (OS) for CD3 positive cells; (c) RFS for CD8 positive cells; (d) OS for CD8 positive cells; (e) RFS for Foxp3 positive cells; (f) OS for Foxp3 positive cells; (g) RFS for α-SMA positive cells; (h) OS for α-SMA positive cells.* $P < 0.05$, ** $P < 0.01$, NS, not significant.

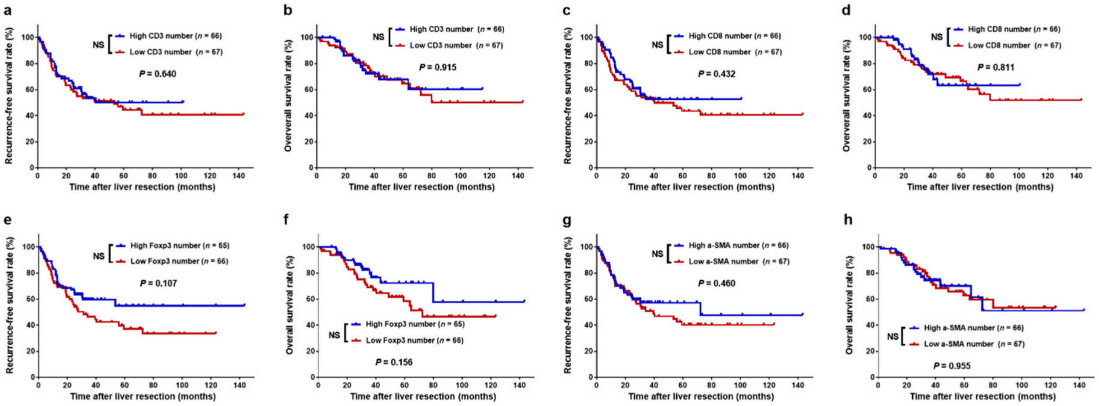

Figure S3. Kaplan–Meier survival curves of CLO patients after liver metastasectomy stratified by the different number of tumour-infiltrating lymphocyte and α-SMA positive cells in peritumoural regions of liver metastases. (a) Recurrence-free survival (RFS) for CD3 positive cells number; (b) overall survival (OS) for CD3 positive cells; (c) RFS for CD8 positive cells; (d) OS for CD8 positive cells; (e) RFS for Foxp3 positive cells; (f) OS for Foxp3 positive cells; (g) RFS for α-SMA positive cells; (h) OS for α-SMA positive cells.* $P < 0.05$, ** $P < 0.01$, NS, not significant.

Table S1. Immunohistochemical multiplex staining protocol.

Antigen	Primary antibody		Catalogue number	Secondary polymer		TSA fluorophore (nm)
	Concentration	Provider		Polymer	Provider	
CD3	1:800	ZSGB-BIO	ZA-0503	EnVision Detection Systems	Dako Cytomation	520
CD8	1:400	ZSGB-BIO	ZA-0508			690
Foxp3	1:100	Abcam	ab20034			620
α-SMA	1:1000	ZSGB-BIO	ZM-0003			570
Pan-Keratin	1:400	CST	4545S			520

Abbreviations: TSA, tyramide signal amplification, CST, Cell Signaling Technology.
